# The degree of bother and healthcare seeking behaviour in women with symptoms of pelvic organ prolapse from a developing gulf country

**DOI:** 10.1186/s12905-018-0570-8

**Published:** 2018-05-30

**Authors:** Fayez T. Hammad, Hassan M. Elbiss, Nawal Osman

**Affiliations:** 1 Department of Surgery, College of Medicine and Health Sciences, United Arab Emirates University, PO Box 17666, Al Ain, United Arab Emirates; 2 Department of Obstetrics and Gynaecology, College of Medicine and Health Sciences, United Arab Emirates University, Al Ain, United Arab Emirates

**Keywords:** Pelvic organ prolapse, Bother, Healthcare seeking behaviour, Emirati women

## Abstract

**Background:**

The healthcare-seeking behaviour of women with pelvic organ prolapse (POP) is affected by several factors including the cultural background. There is limited number of studies which addressed the healthcare-seeking behaviour in women with POP. The aim of this study was to determine the degree of bother, social impact and healthcare seeking behaviour of symptoms of POP in one of the Gulf countries and compare the results to published data from other areas.

**Methods:**

All women who attended the three family development centres in our city between January 2010 and January 2011 and who had symptoms suggestive of POP were included in the study. The data was collected by well-trained interviewers.

**Results:**

One hundred twenty-seven women reported symptoms of POP (mean age: 38.2 years; range: 18–71). Out of these, 111 (87.4%) had at least one activity (physical, social or prayers) or sexual relationship affected by POP symptoms. In 49 women (38%), the effect on at least one of these activities or relationships has been described as moderate and in 18 women (14%), the effect was severe.

Sixty-nine women (54%) did not seek medical advice due to: embarrassment to see medical doctors (51%), the belief that POP is normal among women (51%), hope for spontaneous resolution (48%), embarrassment to see male doctors (33%) and unawareness of the existence of medical treatment (30%).

On univariate analysis, the need to insert the finger in the vagina to empty the bladder or bowel and the interference of symptoms with physical activities, had significantly determined healthcare seeking attitude (*P* < 0.05 for all). However, on multivariate analysis interference with physical activities was the only significant determinant (*P* = 0.04).

**Conclusions:**

Although POP had affected the quality of life in the majority of the affected women, unlike some other societies, more than half failed to seek healthcare advice mainly due to shyness and embarrassment and lack of proper knowledge about the condition. Interference of symptoms with physical activities was the main significant determinants of healthcare-seeking behaviour. Additional teaching campaigns designed according to cultural backgrounds in each society are required to address these sensitive issues.

## Background

Pelvic organ prolapse (POP) is a relatively common condition especially in older women and it might result in very distressing symptoms. Depending on the research methodology and population investigated, wide variation in prevalence has been reported. For instance, on physical examination, some degree of POP is found in 32–98% of women. However, among those who have POP, only 4–8.3% has POP-related symptoms [1–3].

Some studies have suggested that the prevalence of POP and pelvic floor dysfunction, in general, might be affected by race and ethnicity [1, 4, 5]. Indeed, in a previous report we have shown that the prevalence of POP symptoms among women in the Gulf might be slightly higher than other societies [6]. These ethnic and racial variations might influence not only the prevalence and mode of presentation but also the degree of bother and care seeking behaviour of women with different cultural backgrounds.

There is limited number of studies which addressed the healthcare-seeking behaviour in women with symptoms of POP [7–13]. Available research in this field focused mainly on urinary incontinence [14–17] and on pelvic floor dysfunction in general [1, 18, 19]. However, there is evidence to suggest that POP might be associated with higher likelihood of seeking medical advice by the affected women compared to other pelvic disorders [19, 20]. Healthcare seeking behaviour in POP is determined by several factors and reviewing the available studies which addressed the determinants of this behaviour, had shown that obstacles to healthcare seeking can be divided into five main categories:

1. Shyness or embarrassment to speak to healthcare provider/s.
2. Financial factors which affect accessibility to healthcare system.
3. Inadequate knowledge about the diseases such the belief that the disease is part of normal aging, the belief that it will improve on its own or have no successful treatment.
4. Family-related issues such as the lack of support and cooperation of close family members or prioritising family needs over the woman’s own needs.
5. Miscellaneous factors.

Most of the studies which addressed the determinants of this behaviour in women with POP mainly evaluated women from the West [7, 9–12] with some studies addressing this issue in other ethnicities such as Asian women [8, 13]. In the Middle East, although such data is available in some conditions such as urinary incontinence [21], POP-related data is not available for women from the Middle East in general and from the Gulf countries in particular. Therefore, the aim of this research was to study the degree of bother and social impact of symptoms of POP and the determinants of healthcare seeking behaviour in one on the Gulf countries and compare them to published data from other areas. In this study, we investigated women who visited the family development centres in our city. These governmental centres are used essentially for social and cultural activities of women from all social and economic backgrounds.

## Methods

The methods used in this study were as previously described [6]. This study presented the results of a planned analysis of data which was collected at the same time of collecting another data which was previously published and aimed to determine the prevalence and risk factors of symptoms of POP in our population [6]. In summary, all Emirati women who attended all the three family development centres in Al Ain, UAE from January 2010 to January 2011 were included. These centres were the main and only governmental facilities in the city and were visited by large number of Emirati women with different ages and backgrounds. Pregnant women and nulliparous women younger than 30 years were excluded. Written consent was obtained from all eligible participants. The Research Ethical Committee at the College of Medicine and Health Sciences, UAE University approved the study. Written consent was obtained from all eligible subjects.

The data was collected using a questionnaire which was administered by female well-trained interviewers who spoke the same language of the investigated women (Arabic Language). The questionnaire is a custom-designed one. The first part dealt with the basic socio-demographic data. The second part explored the obstetrics, medical and surgical history followed by prolapse-related questions. In this regard, the woman was asked if she felt a dragging lump coming down the vagina, lump coming out of vagina or lump felt or seen outside vagina; the presence of any of these symptoms were considered to indicate the presence of POP in this study. This was followed by other questions to determine the severity of the condition, other vaginal symptoms and if the women had to insert her finger into the vagina to reduce the lump in order to be able to empty the bladder or bowel. Finally, the woman was asked if she had seeked medical advice and if not about the reasons for not doing so. The whole questionnaire was not formally validated but included questions which were used in other studies such as the WHO [22–25]. In addition, and as previously described [6], the questionnaire was tested and re-tested on a pilot female sample to suite our population’s attitudes to discuss the sensitive issues related to POP symptoms. Furthermore, the use of healthcare providers to question the participants and fill the questionnaire eliminated the potential misunderstanding of some of the questions by the participants.

Data were analysed using SPSS version 19.0 (IBM, Armonk, NY, USA). Inter-group comparisons were performed using the Student t test for continuous variables and the chi-square or Fisher exact tests for categorical variables. Multivariate binary logistic regression analysis was performed to determine independent factors which were associated with healthcare seeking behaviour. A *P* value of < 0.05 was considered statistically significant.

## Results

As previously described [6], out of 482 women who were approached and met the inclusion criteria, 429 (89.0%) consented to fully participate in the study. Out of these, 127 women (29.6%) reported symptoms of POP with mean age of 38.2 years (range: 18–71).

**Fig. 1:**
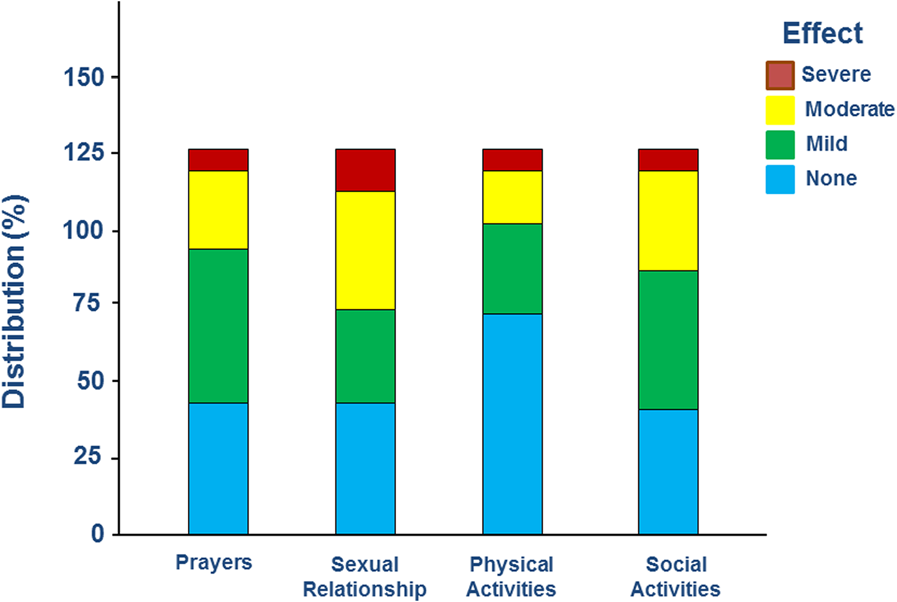
The effect of symptoms of POP on the various activities and relationships

Out of the 127 women, symptoms of POP affected various activities and relationships as follows: physical activities (*n* = 91, 72%), social gathering and activities (*n* = 69, 54%), ability to pray (which involves several body movements) (*n* = 84, 66%) and sexual relationship (n = 91, 72%). The symptoms of POP had affected at least one activity (physical, social or prayers) or sexual relationship in 111 (87%) women. As demonstrated in Fig. 1, these effects ranged from mild to severe. In 44 women (35%), the effect on at least one of these activities or relationship has been described as mild, in 49 women (38%), it was moderate and in 18 women (14%), the effect was severe.

As demonstrated in Table 1, 69 out of the 127 women (54%) did not seek healthcare advice due to different reasons such as the embarrassment to see a medical practitioner in general or a male practitioner in particular and the lack of adequate knowledge about the disease such as the belief that it is normal among women especially older women or the unawareness of the existence of medical treatment.

**Table 1:** Reasons for not seeking medical advice. % represents the percentage of women among the 69 women who did not seek medical advice. Some women might have reported more than one reason

Reason for not seeking a medical advice	No (%)
Embarrassment to see a medical doctor of any gender	35 (51%)
Belief that POP is normal among women especially older women	35 (51%)
Hope for spontaneous resolution of the POP	33 (48%)
Embarrassment to see male doctor	23 (33%)
Unawareness of the existence of medical treatment	21 (30%)

Table 2 describes the socio-demographic determinants of healthcare seeking behaviour. Using univariate analysis, women who seeked medical advice were significantly younger than those who did not (40.1 ± 1.4 vs. 36.6 ± 1.2 years, *P* = 0.03). However, occupation, monthly income and level of education did not determine the healthcare-seeking behaviour (*P* > 0.05 for all).

**Table 2:** Socio-demographic determinants of healthcare-seeking behaviour for women with symptoms of POP. All variables are presented by crude number apart from Age which was presented by mean ± standard error of the mean. *P* value indicates statistical significance according to univariate analysis

	Non-seeking group	Seeking group	*P* Value
Number	69	58	
Age (years)	36.6 ± 1.2	40.1 ± 1.4	0.03
Monthly Income (Dirham)			0.67
< 5000	3	2	
5000–10,000	31	22	
> 10,000	35	34	
Education			0.82
University	8	5	
Secondary school	18	15	
Primary school	26	26	
Illiterate	17	12	
Occupation			0.96
Housewife	49	42	
Office job	11	8	
Physical	9	8	

Table 3 demonstrates the determinants of healthcare seeking behaviour according to the symptoms or manoeuvres which were performed to relieve the symptoms. The degree of vaginal soreness did not affect the seeking behaviour. However, the need to insert the finger into the vagina to empty the bladder or bowel was significantly associated with seeking medical advice on univariate analysis (*P* < 0.05 for both).

**Table 3:** Determinants of healthcare-seeking behaviour for women with symptoms of POP according to the reported symptoms. *P* value indicates statistical significance according to univariate analysis

	Non-seeking group	Seeking group	*P* Value
Number	69	58	
Awareness of vaginal soreness			0.19
Never	22	12	
Very occasional	33	31	
Sometimes	6	11	
Most of times	5	1	
All the time	3	3	
Insertion of finger into vagina to start or complete bladder emptying			0.04
Never	52	32	
Very occasional	10	14	
Sometimes	3	9	
Most of times	1	2	
All the time	3	1	
Insertion of finger into vagina to start or complete bowel emptying			0.01
Never	51	28	
Very occasional	11	20	
Sometimes	3	8	
Most of times	2	1	
All the time	2	1	

The effect of POP symptoms on various activities and relationships and their relation to the seeking behaviour was demonstrated on Table 4. Only interference with physical activities was significantly associated with seeking medical advice on univariate analysis (*P* = 0.005).

**Table 4:** Determinants of healthcare-seeking behaviour for women with symptoms of POP according to the interference with various activities and relationships. *P* value indicates statistical significance according to univariate analysis

	Non-seeking group	Seeking group	*P* Value
Number	69	58	
Interference with Prayers			0.14
No interference	25	18	
Mild	27	27	
Moderate	16	8	
Severe	1	5	
Interference with sexual relationship			0.17
No interference	20	16	
Mild	23	15	
Moderate	16	23	
Severe	10	4	
Interference with social activities			0.14
No interference	33	25	
Mild	20	26	
Moderate	14	5	
Severe	2	2	
Interference with Physical activities			0.005
No interference	27	9	
Mild	20	31	
Moderate	20	14	
Severe	2	4	

On multivariate analysis of all the factors which were significantly associated with seeking medical advice on univariate analysis only interference of POP symptoms with physical activity independently predicted seeking medical advice in our study [β: 0.781, SE: 0.372, Exp(β): 2.156, *P* = 0.04]. The remaining factors did not independently predict seeking behavior (age: β: 0.081, SE: 0.02, Exp(β): 1.018, *P* = 0.4; insertion of finger to empty bladder: β: -0.128, SE: 0.602, Exp(β): 0.880, *P* = 0.8; insertion of finger to empty bowel: β: 0.835, SE: 0.597, Exp(β): 2.302, *P* = 0.2).

## Discussion

In this study, we have shown that the vast majority of women who reported POP symptoms were bothered by the condition and approximately half of those affected were moderately or severely bothered by the symptoms. We have also demonstrated that more than half of the affected women did not seek medical advice for various reasons. To the best of our knowledge, this is the first study which assessed the degree of bother, social impact and healthcare seeking behaviour in women with POP symptoms from this geographical region.

The degree of bother and social impact of symptoms of POP in the current study is similar to those reported from the West [10, 26]. In a population-based study on American women, Rortveit and colleagues reported that almost 50% of women with POP symptoms had moderate or great distress and in 35%, the POP symptoms affected at least one physical, social or sexual activity [26]. Similar finding were reported in a similar study from Nepal [13]. Collectively, these studies and our study indicate that regardless of the cultural background, women in various nations appear to be bothered and socially well-affected by symptoms of POP. The current study, however, indicates that women in our society are less likely to seek medical advice compared to women from the West. For instance, in a population-based study, Morrill et al. showed that 73% of American women had seeked medical advice compared to only 46% in our study [20].

The obstacles to healthcare seeking in our study are different from those reported from other communities. For instance, in our study, financial factors including insurance concerns were not important in determining whether an affected woman would seek medical advice because all individuals in our society have free access to healthcare system at various levels from primary healthcare to tertiary centres. This is in contrast to some other studies in which financial issues were of major concern and actually prevented women from seeking advice despite the need to do so [8, 19].

In our society, shyness or embarrassment to discuss POP symptoms with healthcare providers of any gender and male providers in particular appears to be one of the strongest determinants of seeking behaviour. This is probably due to local culture-related factors and has only been reported by limited number of studies [8].

The other important determinant of the seeking behaviour in our society is the lack of adequate knowledge about the condition such as the belief that the disease is part of normal aging and the unawareness of the existence of medical treatment. Such a belief appears also to be common across other societies [8, 13, 18, 19] and obviously, such thinking would discourage women from seeking medical advice.

In contrary to some other societies, family-related issues and the fear of lack of support of family members did not appear to be an important issue in our study [8, 13, 18]. Probably this is due to the nature of family structure in our area. Most families are extended large families which provide extra-support to its members. As a whole, the findings of the current study and previous studies in this field indicate that not only the percentage of women who seek medical advice but also the factors which influence this behaviour vary widely among different societies.

The main factor which pushed women to seek medical advice in this study was the interference of the POP symptoms with daily physical activities. Interfering with physical activities would seriously compromise women’s ability to meet their personal and family commitments and might also prevent them from undertaking certain professions.

The relationship of age to the healthcare seeking behaviour has been investigated previously with conflicting results. Some studies which investigated healthcare seeking behaviour in pelvic floor disorders in general have shown that older women had lower threshold to seek medical advice compared to younger women [19, 20]. On the other hand, in another study which addressed the seeking behaviour in POP, younger women were found to be more likely to seek medical advice [7]. Using multivariate analysis, our results clearly demonstrated that age was not a significant determinant of healthcare seeking behaviour. These discrepancies could probably be due to cultural differences and attitudes towards disease tolerance and seeking help from others in general.

To our surprise, the level of education has not been shown in our study or in other similar studies [7, 19, 20] to determine the healthcare seeking behaviour in women with POP symptoms. Cultural background and attitudes appear to be stronger factors which determine healthcare seeking behaviour across different societies regardless of the educational level.

The current study has some limitations. So, although it is not strictly a whole population-based study, it included all women from the three family development centres who met the study criteria. The visitors of these governmental centres are women of different ages and from different social and cultural backgrounds and these centres are essentially used for variable educational, social and cultural activities. They are not part of healthcare system and therefore, not used for seeking medical care. Hence, the study population in this research is expected to be better representing the general population compared to other studies which used patients from hospitals or primary healthcare centres [9–12]. The other potential limitation is the inclusion of women from only one city. This renders the data difficult to generalise to the whole country. However, since approximately one fifth of the whole Emirati population lives in this city [27, 28], the results obtained might be a reasonable reflection of the whole country.

One of the other limitations of the study is the lack of vaginal examination which would have been very difficult in this conservative society. However, it is unlikely this would have affected the results of this study as good correlation has been found to exist between the symptoms with the presence of anatomical POP on physical examination [29]. In the present study, symptoms of POP were identified by healthcare providers who administered the questionnaire. This might have caused some bias compared to self-administered questionnaires. Nevertheless, the use of healthcare providers had enabled us to investigate illiterate women and decreased the potential misinterpretation of these sensitive questions by some of the participants.

Finally, the current study and other similar ones should encourage further campaigns to educate women about pelvic floor disorders including POP. Such campaigns and educational sessions have been shown to improve the general knowledge of women about this condition [30] and should tackle the specific obstacles to healthcare seeking in that particular society. In our society, these campaigns should be coordinated by a combination of well-trained female healthcare providers and social workers who are able to tackle not only health issues but other sensitive issues such as shyness and cultural-related obstacles.

## Conclusions

Although symptoms of POP had affected the quality of life in the majority of the affected women in our society, unlike some other societies, more than half failed to seek healthcare advice. This was mainly due to shyness, embarrassment to speak to healthcare providers and lack of proper knowledge about the condition. Interference of symptoms with physical activities was the main significant determinants of healthcare-seeking behaviour. Additional teaching campaigns designed according to cultural backgrounds in each society are required to address these sensitive issues.

## Abbreviations

POP: Pelvic organ prolapse
UAE: United Arab Emirates
